# Prediction Model for Physical Activity Level in Primary School Students

**DOI:** 10.3390/ijerph19052987

**Published:** 2022-03-04

**Authors:** Myriam Alvariñas-Villaverde, Javier Martínez-Torres, Belén Toja-Reboredo, Miguel González-Valeiro

**Affiliations:** 1 Department of Special Didactics, Faculty of Education and Sport Sciences, University of Vigo, 36310 Vigo, Spain; myalva@uvigo.es; 2 Research Group on Education, Physical Activity and Health (GIES10), Galicia Sur Research Institute (IIS Galicia Sur), SERGAS-UVIGO, 36312 Vigo, Spain; 3 Department of Applied Mathematics I, Telecommunications Engineering School, University of Vigo, 36310 Vigo, Spain; javmartinez@uvigo.es; 4 Department of Physical and Sports Education, Faculty of Physical Education and Sport Sciences, University of A Coruña, 15001 A Coruña, Spain; belen.toja@udc.es

**Keywords:** decision trees, predicting variables, perceived motor competence, friendships, gender, student’s perceptions

## Abstract

The aim of this study was to provide an explanatory prediction model for physical activity level in children, involving a number of influencing variables. In total, 1971 people participated in the study: 657 primary school students and their respective fathers and mothers from 15 schools of Galicia (Spain). The International Questionnaire on Physical Education, Health and Lifestyle was administered. The findings revealed that school year, sex, physical perceived competence and sport practice with friends had a statistically significant relationship on physical activity index. By contrast, the association between the weekly participation of fathers or mothers in sports in the practice of children could not be confirmed. Sport practice with friends was the main predicting variable for physical activity level. Physical perceived competence showed great relevance as well. This knowledge could be of interest to help increase adherence to practice and preventing withdrawal, helping students to stay active and acquire healthy habits for the future.

## 1. Introduction

Over the past few years, a relationship between physical activity (PA) and learning has been suggested [1,2]. This connection between physical activity and learning is nowadays sufficiently shown from the evidence of neuroeducation [3]. 

Similarly, special attention has been paid in the literature to the study of various factors that affect children’s PA [4,5,6]. The variable sex (and its implications in terms of gender) is paramount when studying physical and sport behaviour. The physical activity level (PAL) is higher in boys than in girls [7,8]. It is currently considered to be one of the variables to show correlation with PA in a largest number of studies [9]. This has been widely explained in the literature in terms of gender, sport socialisation and their implications related to stereotypes, motivation and differentiated behaviours in this context [10,11].

Another widely analysed variable is age (or, in some cases, the school year). PA levels tend to decline with age from childhood through adolescence [12,13]. This transition period reflects a regression in PA practice [14] and a higher risk of being overweight or obese [15]. For this reason, great attention must be paid to physical and sporting behaviour at the end of primary school, just before beginning the secondary school. This will help prevent regression in PA and avoid sedentary behaviours.

Additionally, perceived physical competence (PPC) is regarded to be a cognitive variable with important motivational effects. Numerous studies have brought this relationship to light, showing a positive association between PPC and practice level [16,17]. Likewise, this construct is directly related to gender issues, since individuals perceive themselves more capable of doing an activity if it is associated with the socially established roles [18]. Thus, individuals are either encouraged to practise or discouraged from practising PA. Regarding age, Zhang et al. [19] explained that this construct starts gaining importance at the age of 10 through the socialisation with peers and the information gathered from coaches and teachers.

Moreover, if we focus on the social agents that have influence on practice, friends seem to have great relevance [20]. It has been showed through accelerometry that the presence of a friend increases PA behaviour already in the first years of life [4]. This relationship on PA behaviour over time has also been confirmed in students between 9 and 11 years old [21]. More specifically, the variable sport practice with friends (SPF) or participation in physical activity with friends has shown positive association with activity level in primary school students [22,23,24]. 

Similarly, the importance of other agents, such as family, on this behaviour has been analysed, giving special relevance to parental support as a potential endorsing factor for their children’s practice. When focusing on a primary school context, studies such as the one conducted by Bringolf-Isler et al. [25] reveal the existence of a strong correlation in moderate-to-vigorous physical activity between parents and children from 10 to 12 years old. Eriksson et al. [17] and Jiménez-Pavón et al. [26] found the same association. It has even been shown in a longitudinal study that PA of parents during pregnancy and the beginning of the child’s life is associated with the PA level of the child at the age of 11–12 [27]. Nevertheless, the specific association of parental practices on child’s PA remains to be inconclusive [9,28]. Moreover, these relationships have been found to be minimal in some meta-analysis studies [6,29].

The studies that have revealed associations between the sport behaviour of parents and their children have also reported differences based on gender. Thus, it has been indicated that mothers’ physical activity was associated with children’s physical activity, but no such association was found between fathers and children [30]. However, previous research has also suggested that fathers have the greatest influence. For instance, Davison et al. [31] found that mothers provided important support, but more at a logistic level, whereas fathers provided more explicit support, acting as a model to their children.

Given the relevance of these aspects and the relationships found in previous research, it becomes necessary to establish some patterns to predict whether an individual will be active or inactive, since this type of study is scarce. Therefore, the first aim of this study was to analyse the variables that affect the physical activity index (PAI) of primary school students. Furthermore, the second aim was to provide an explanatory prediction model for the physical activity level (PAL), using a number of potentially influencing variables.

## 2. Materials and Methods

### 2.1. Participants

The participants (*n* = 1971) took part in the study. From them, 657 were students of fifth and sixth year (331 boys and 326 girls) aged between 10 and 14 years old (M = 11.15, SD = 0.908). They studied in 15 selected schools from Galicia (Spain), chosen by sampling in the seven main urban areas in that region. In every school, the group with the highest number of students was selected from every year. The sample was completed with the students’ parents: 657 fathers and 657 mothers.

### 2.2. Instrument

The International Questionnaire on Physical Education, Health and Lifestyle was administered. There are two versions of this instrument: one for parents and one for students. They had been validated by Mourelle [32] for Spanish population.

The Finnish PA index was applied in order to calculate the PA performed by the students. This is representative of the probability of carrying out physical activity in the future. This has been confirmed by several longitudinal studies on the same participants over 20 years [33]. It results from adding up five items on a four-point scale: weekly PA practice, participation in non-organised PA, participation in organised PA, weekly hours of vigorous practice and participation in sport competitions. These items had been used with other European populations, yielding good reliability values [24,34].

The variables used in this study were:

- PAI: describes the activity level of every student and its value ranges between 5 and 20. The variable PAI was recoded twice. Firstly, the sample was divided into three groups, as explained in the study by Mourelle [32]. This recoding divided the participants into low (PAI below 9), medium (PAI between 9 and 13) and high PAL (PAI above 13). Moreover, and due to research requirements, the sample was divided into two groups following Marques et al. [24], who suggested that the sample median should be taken at threshold value. In this case, the cut-off point was set at PAI = 12: less active or sedentary (scores equal to or below 12) and active (scores equal to or above 13).
- PAL Classification 1—(PAL1): category in which a participant was included after the division into low, medium and high level.
- PAL Classification 2—(PAL2): category in which a participant was included after the division into active and less active or sedentary.
- Sex: indicates whether the person was male or female.
- Year: indicates whether the participant was in their 5th or 6th year of primary school (5th Y-PS or 6th Y-PS).
- PPC: indicates the participant’s perceived competence in sport: do not know, good, above average, average, below average, poor.
- SPF: describes how often the participant practised sport with their friends: do not know, never, sometimes, often, usually, always.
- MWPS: describes the mother’s weekly participation in sport. Its values range from 0 to 7 (7 meaning 7 times per week or more).
- FWPS: describes the father’s weekly participation in sport. Its values range, as in the previous case, from 0 to 7.
- Thus, a classification model for the variable PAI in its two versions (PAL1 and PAL2) was established with this set of variables. The rest of the variables are explanatory variables whose statistical association on the dependent variable will be analysed.

### 2.3. Procedure

The procedure started by the manual recoding of questionnaires and envelopes, assigning a code to each of them with the aim to identify every student by their parents/legal guardians, school, year and city. These questionnaires, introduced by a cover letter, were administered to the students at school. The one for fathers/mothers was distributed and it was collected back within one or two weeks (depending on the school calendar).

Once the informed consent had been signed, the questionnaire was administered by members of the research group in the presence of the Physical Education teachers.

The protocol was approved by the University of La Coruña. For the studies conducted in Portugal, the approval of the ethics committee of the Faculty of Human Kinetics (University of Lisboa) and the Portuguese Ministry of Education was obtained. These studies are part of a global initiative from the Euro American Network of Physical Activity, Education and Health.

### 2.4. Statistical Data Analysis

**Figure 1:**
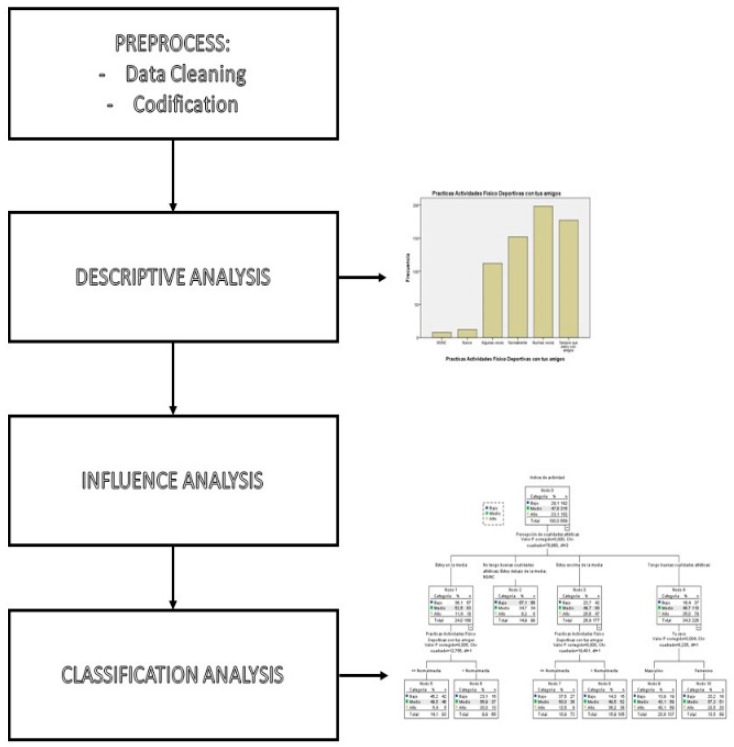
Diagram of the data analysis process.

Once the data were collected, introduced in the computer, cleaned (missing values and outliers determined) and analysed for inconsistencies, various statistical analyses were conducted, divided into several phases (Figure 1).

Firstly, a descriptive analysis was performed to gain general knowledge on the sample through the calculation of measures of position, dispersion and shape. By doing so, the first data were obtained regarding homogeneity and outliers that may have caused incongruous results.

During the influential analysis phase, a one-way analysis of variance (ANOVA) was conducted to study the association with several nominal variables on PAI.

In the last analysis phase, it is important to note that the dependent variable showed both quantitative and qualitative aspects once it was grouped. Therefore, the classification and regression trees (CART) were chosen as the best technique, due to their usefulness when drawing conclusions:

- Error rate as classification model. The error rate was analysed in a validation group (10% of total sample).
- Classification rule for every observation. Every observation (participant) was introduced into the model from the top and the tree was read downwards depending on the variable values, until the final point was reached and the percentage for each class was obtained.
- Importance of the variables in the prediction of the variable under analysis. Depending on the position of the variables (higher or lower in the model), they were given certain relevance within the model classification rule.
- Determination of the probability of belonging to each PAL class, based on the predicting variables for every participant. By analysing the highest values for each class at the terminal tree points (“leaves”), the classification rule can be established.

Lastly, the confidence interval for PAI was calculated, providing the mean PAL for the whole population with a specific probability. All the analyses were conducted using the statistical package SPSS 21.0 (SPSS, Chicago, IL, USA).

## 3. Results

### 3.1. Descriptive Analysis

The descriptive analysis of the variables showed that the percentage of boys and girls in the sample was almost equal (50.4% and 49.6%, respectively). The number of children who were in their fifth and sixth year was also very similar (25.1% and 28.2%, respectively).

Regarding the PAL of the students, it was observed that only 23.1% of the participants were classified into the highest level. A total of 29.1% were included in the lowest level and almost half of the students belonged to the intermediate level.

With regard to PPC, it was noteworthy that a considerable number of participants reported to have good competence (34.1%), or at least above average (26.9%). The percentage of students who reported to be below average or to not have good competence stayed under 13%. 

In addition, it was found that most of the students engaged in physical activity with their friends. Eighty percent did so often, usually or always. Only 1.8% did not.

It must be highlighted that mothers’ and fathers’ weekly participation in sport (MWPS and FWPS) was nonexistent in 43% to 48.1% of the cases, respectively.

### 3.2. Influential Analysis

The next step was to determine whether there was a relationship between each independent variable and the variable under study. The ANOVA tests revealed that the variables that had a statistically significant effect on PAI (*p* < 0.05) were school year (F = 5.736, *p* = 0.017), sex (F = 30.818, *p* < 0.0001), PPC (F = 19.728, *p* < 0.0001) and SPF (F = 15.702, *p* < 0.0001). No significant differences were observed in the variables FWPS (F = 1.294, *p* = 0.250) and MWPS (F = 0.989, *p* = 0.438) among groups, so their association with PAI could not be confirmed. 

### 3.3. Predictive Analysis

**Figure 2:**
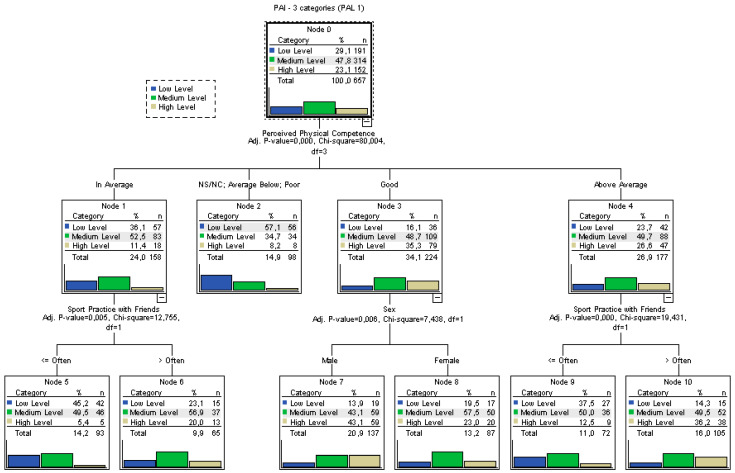
Classification tree for the variable PAL1.

As mentioned above, the dependent variable showed both quantitative and qualitative aspects once it was grouped. In fact, it should not be analysed as a quantitative variable since it may take values from 5 to 20, but not any numerical value. For this reason, it was decided to use classification techniques. In this study, classification trees were built using PAL1 (PAI divided into three categories) and PAL2 (PAI divided into two categories) as dependent variables. The first tree was built for variable PAL1 (Figure 2).

The main results that can be extracted from analysing the tree are:

- The most important variable when categorising PAL was found to be PPC, as it was the highest variable in the tree.
- The variables that did not show associations according to the ANOVA or the correlations were confirmed by this model, since they were not included in any specific level of the tree. This showed the consistency of the analyses.
- The most likely individuals to present low PAL (57.1%) were those with very low PPC.
- Apart from those with low PPC, the most likely participants to have low PAL were those who perceived their physical competence as “average” and practised sports with their friends less than “often”. The probability of belonging to this group was 45.2%.
- The most likely individuals to have high PAL were boys with good PPC. Their probability to present high PAL was 43.1%.
- Any classification rule can be extracted for the three PA types, apart from the extreme cases analysed above.
- For the variable SPF, it is clear that one group could be created with individuals who practised sport less than “often” and one with those who practised it with higher frequency. This brings to light that the variable was divided into too many categories and a binary classification would be enough, using that category as a discriminating factor.
- The tree classification model yielded an error rate of 40%.

**Figure 3:**
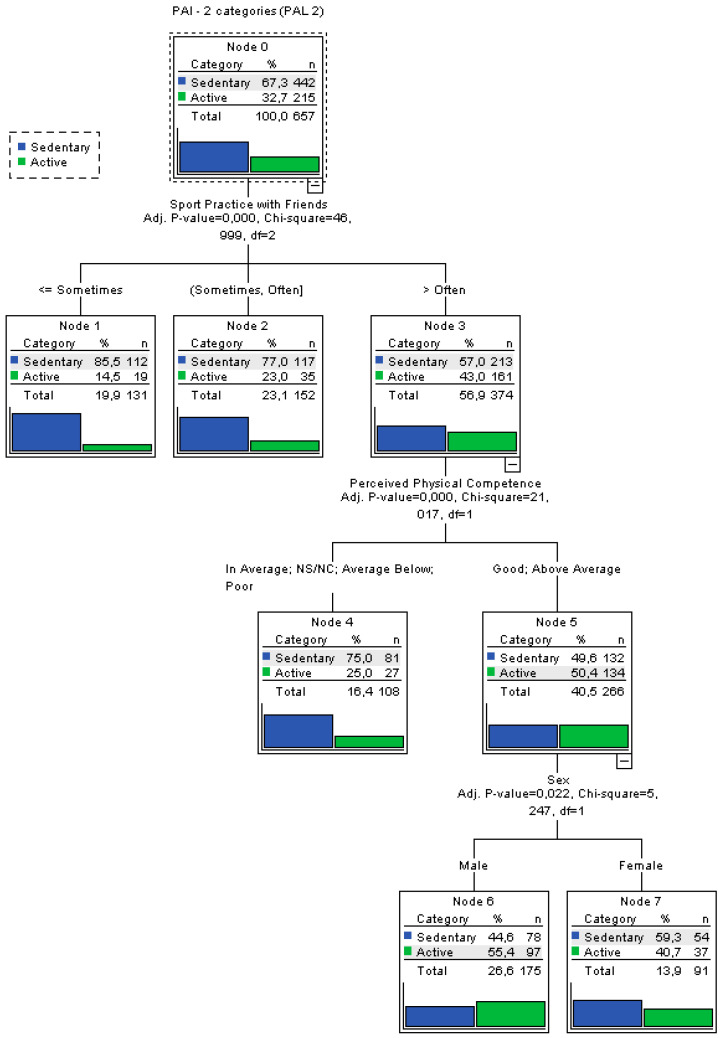
Classification tree for the variable PAL2.

A classification tree was built for variable PAL2 (Figure 3) (both classes are shown as sedentary, equivalent to sedentary or less active and active), since it presented higher homogeneity among the participants analysed (58% and 42%).

The main results that can be obtained from analysing the tree are:

- The most relevant variable when categorising PAL was found to be SPF, as it was the highest variable in the tree.
- The variables that did not have influence according to the ANOVA or the correlations were confirmed by this model, since they were not included in any specific level of the tree. This showed the consistency of the analyses.
- This analysis revealed that the variable sex did not affect the classification.
- The most likely individuals to be less active (85.5%) were those with very low SPF.
- The most likely individuals to be active were those who showed good PPC, usually practised sports with their friends. Their probability to present high PAL was 55.4%.
- Any classification rule can be extracted for the three PA types, apart from the extreme cases analysed above.
- For the variable SPF, it is clear that one group could be created with individuals who practised sport with their friends less than “often” and one with those who practised it with higher frequency. This brings to light that the variable was divided into too many categories and a three classification would be enough, using that category as a discriminating factor.
- The tree classification model yielded an error rate of 29.5%.

Lastly, the confidence interval for PAI was calculated with a confidence level of 95% (10.40, 10.91). This indicates that the mean value for the whole population would fall into that interval with a probability of 95%. Consequently, the average physical activity level can be described as medium when using three categories and as less active or sedentary when using two categories.

## 4. Discussion

The purpose of this study was to analyse the variables that can be related to the PAI of primary school students. Moreover, it aimed to provide an explanatory prediction model for PAL, producing classification rules for the participants.

Significant differences were found based on sex, PAI being higher in boys than in girls. This fact has not changed in the past few years, as confirmed by studies in several contexts [8,15,25].

Students from the sixth year were observed to have higher PA scores than those from the fifth year. It has been clearly shown in the literature that the PA level decreases with age and school year, especially with the change from primary to secondary school [12,13]. However, some studies have reported these correlations with PA to be inconsistent at earlier ages [5]. Our data support this idea, since the students from the higher school year obtained higher scores.

As expected, the association between PPC and PAI turned to be very relevant. In the last few decades, the importance of this variable in physical activity contexts has been suggested. Thus, those individuals with better PPC presented higher PAL, and vice versa [16,17,35].

With regard to the variable SPF, our results were in line with previous research. For example, Marques et al. [24] found that the most active children between 10 and 12 years old showed higher scores in this item. Furthermore, Finnerty et al. [22] confirmed the existence of significant positive correlations between PA levels and friends taking part in PA or exercise with the participant in students aged 9 to 13. This effect of practising with friends has also been verified when studying PA over time [21]. Specifically, Coppinger et al. [21] investigated PA and dietary intake of children aged 9–11 years, and the influence of peers on these behaviours over a two-year period. The findings revealed that throughout the survey, the behaviour of peers influenced the PA that the children engaged in.

In agreement with previous findings, our results revealed that the physical activity of parents was independent from their children’s PA level [5,28]. A meta-analysis showed that parental modelling was weakly associated with a child’s PA [6].

By contrast, other important studies did confirm the existence of significant relationships between parents’ and children’s PA. For example, ENERGY data revealed that, overall, parental modelling of PA showed significant associations with a child’s PA [26]. Bringolf-Isler et al. [25], using data from SOPHYA-study regarding children of the same age, also found strong correlations between parents’ and children’s PA. The sociocultural factor was highlighted in both aforementioned studies. Likewise, the relationship with the mother [17,30] or the father [17,31] on children’s PA level was considerable in some studies. The parental modelling of PA had greater or smaller weight depending on the country or region under study. According to this, it is essential to design and implement interventions for specific subgroups.

The first classification tree, built for PAL1, revealed that PPC was the most important variable in the categorisation. This first approach brought to light the relevance of practising with friends in order to predict low PAL. 

The error rate obtained in the first tree motivated the creation of a second model using the variable PAL2, which allowed for a better explanation of PAL. The dominant variable was SPF and the parent-related variables were not relevant. Lastly, sex did not affect the classification.

Both classification trees evidenced the power of SPF to predict PA. In agreement with the present research’s findings, other studies have shown the tendency of this variable to predict physical activity practice. Kirby et al. [23] conducted a longitudinal study and observed this influence to be significantly associated with being active in primary school students. The relevance of this aspect has also been confirmed in an important review that analysed different influencing elements related to friends [20]. More specifically, they concluded that, in 9 out of 10 studies, participation with friends and the presence of friends during PA was associated with the participants’ PA. It must be noted that one inclusion criterion of this study was the existence of a PA-predicting variable.

Given the relevance of practising physical activity with friends, and its relationship with being more or less active, it seems necessary to involve these groups of friends in PA promotion projects. 

As explained above, PPC has been associated with PAL in several studies. In fact, its predicting power regarding enjoyment and practising behaviour has been shown, as it happened in the present study [19,36,37].

Additionally, Burkhalter and Wendt [36] concluded that, independent from sex, the psychological variables of PPC toward fitness and alienation were related to physical performance. Thus, highly alienated students and children with lower PPC were less fit. 

These findings provide education and health practitioners with valuable information on the importance of improving PPC in all students from early ages. Strategies that enable children to perceive themselves as more competent and confident of their capacities can be implemented in formal environments, but also in non-formal or informal contexts. In this regard, it is essential to: offer a wide variety of activities and to encourage enjoyment in all of them, propose different levels of problem resolution in order to provide rewarding experiences, provide positive feedback, help students make adaptive causal attributions based on effort, break gender stereotypes and stigmas, etc. By doing so, PPC will improve, students’ adhesion to PA will increase and their lifestyles will become healthier.

In terms of limitations, it must be noted that the present study was conducted in an urban context, which prevented it from getting an exhaustive view of the practising behaviour. Furthermore, data collection was cross-sectional and the measure of PA was self-reported. In future studies, it would be desirable to include rural populations and to use instruments such as accelerometers. Furthermore, it would be interesting to check whether the associations and prediction models still apply in longitudinal research with a larger sample. It also seems necessary to gain knowledge on the optimal mechanisms, contents, didactic methods and strategies to increase PPC and SPF in sport and physical education contexts. Likewise, it is suggested to conduct intervention studies involving friends in order to observe which elements are crucial to create and maintain sport socialisation.

## 5. Conclusions

The findings of this study contribute to the increase in knowledge on the factors that affect PAL in primary school students. Particularly, the analysis through classification trees has allowed for the extraction of classification rules and prediction variables of practice levels (active vs. less active/sedentary participants). SPF was the most important prediction variable, although PPC was found to be very relevant as well. This knowledge could be of interest to help increase adherence to practice and preventing sport dropouts, helping students to stay active and acquire healthy habits for the future.

## Data Availability

The data are not publicly available due to confidentiality reasons.
